# P-1711. Attributable and Contributable Mortalities of Culture-Positive Invasive Aspergillosis in Unselected Patients with Hematologic Malignancies at a Tertiary Care Cancer Center: No Room for Complacency

**DOI:** 10.1093/ofid/ofaf695.1883

**Published:** 2026-01-11

**Authors:** Ariel D Szvalb, Teny John, Sebastian Wurster, Takahiro Matsuo, Ying Jiang, Dimitrios P Kontoyiannis

**Affiliations:** The University of Texas MD Anderson Cancer Center, Houston, TX; The University of Texas MD Anderson Cancer Center, Houston, TX; The University of Texas MD Anderson Cancer Center, Houston, TX; Mayo Clinic, Rochester, MN; The University of Texas MD Anderson Cancer Center, Houston, TX; The University of Texas MD Anderson Cancer Center, Houston, TX

## Abstract

**Background:**

Invasive aspergillosis (IA) remains a common cause of both direct and contributable mortality in patients with hematologic malignancies (HM). Given the advances in antifungal management, it would be important to assess IA-attributable mortality (AM) and contributable mortality (CM), in addition to overall mortality (OM).

**Methods:**

We retrospectively reviewed the records of 76 consecutive HM patients with proven/probable culture-positive IA (MSG/EORTC criteria) from 2015 to 2021. Death causality was adjudicated based on published criteria. AM was defined as death “due to” IA, while CM as death “due to” and “with” IA. OM encompassed death from any cause. Mortality rates and death causalities were evaluated at 2-, 4-, 6-, and 12-week intervals following IA diagnosis. A multivariate competing risk analysis was performed to identify independent risk factors for 6-week AM, with other death causalities as the competing event. To increase diagnostic accuracy, we excluded culture-negative, *Aspergillus* galactomannan (GM) positive cases, as GM can be produced by other hyalohyphomycetes.

**Results:**

The median patient age was 60 years (61%). Lymphoma and multiple myeloma (L/MM) were the most common HM (40, 53%). The remaining patients had active leukemia. Twenty-nine patients (38%) had prior stem cell transplantation, and 10 (13%) had graft-versus-host disease. *Aspergillus fumigatus* was the most common species (47%). Six and 12-week mortality rates were 18.7% and 22.7% for AM, 42.7% and 49.3% for CM, and 45.3% and 53.3% for OM, respectively. Notably, 2-week OM was 27.6%. Lack of neutrophil recovery was the only independent risk factor for 6-week AM on multivariate analysis (OR=4.2, p=0.006).

**Conclusion:**

Our findings reflect the modern reality of culture-documented IA, occurring mostly in HM patients with heavily pretreated L/MM not routinely receiving mold-active prophylaxis, and in those with active leukemia. In this real-life contemporary HM population, culture-positive IA was associated with high cumulative OM and CM. Understanding AM and CM rates could lead to more accurate sample size estimations in clinical mycology trials. Such trials should be pragmatic and assess antifungal failure at an earlier time point than the traditional 4-week mark post-diagnosis.

**Disclosures:**

Dimitrios P. Kontoyiannis, MD, AbbVie, Inc: Advisory Board Participation|Astellas: Grant/Research Support|Basilea: Advisor/Consultant|Cidara, Inc: Advisory Board Participation|F2G, Inc: Advisor/Consultant|Gilead: Advisor/Consultant|Gilead: Grant/Research Support|Knight, Inc: Honoraria|Pfizer: Advisor/Consultant|Scynexis: Advisor/Consultant|TTF Pharmaceuticals, Inc: Advisor/Consultant|US-Israel Binational Sci Foundation: Grant/Research Support

